# Combined First Month Body Weight Loss and Development of Tolerance as Predictors of 6-Month Efficacy of Mazindol in Mild and Moderate Obese Subjects

**DOI:** 10.3390/jcm11113211

**Published:** 2022-06-04

**Authors:** Juan Carlos Huerta-Cruz, Héctor Isaac Rocha-González, Ashuin Kammar-García, Samuel Canizales-Quinteros, Lina Marcela Barranco-Garduño, Juan Gerardo Reyes-García

**Affiliations:** 1 Unidad de Investigación en Farmacología, Instituto Nacional de Enfermedades Respiratorias, Ismael Cosio Villegas, Secretaría de Salud, Calzada de Tlalpan 4502, Col. Belisario Domínguez Sección XVI, Tlalpan, Mexico City 14080, Mexico; pharman007@hotmail.com (J.C.H.-C.); linamarcelaba@yahoo.com.mx (L.M.B.-G.); 2 Sección de Estudios de Posgrado e Investigación, Escuela Superior de Medicina, Instituto Politécnico Nacional, Plan de San Luis y Díaz Mirón s/n, Col. Casco de Santo Tomas, Miguel Hidalgo, Mexico City 11340, Mexico; heisaac2013@hotmail.com (H.I.R.-G.); kammar_nutrition@hotmail.com (A.K.-G.); 3 Dirección de Investigación, Instituto Nacional de Geriatría, Anillo Periférico 2767, San Jerónimo Lídice, La Magdalena Contreras, Mexico City 10200, Mexico; 4 Facultad de Química, Universidad Nacional Autónoma de México, Circuito Escolar s/n, Ciudad Universitaria, Coyoacán, Mexico City 04510, Mexico; cani@unam.mx; 5 Instituto Nacional de Medicina Genómica, Periférico Sur 4809, Arenal Tepepan, Tlalpan, Mexico City 14610, Mexico

**Keywords:** interindividual variability, mazindol, obesity, tolerance, weight loss efficacy

## Abstract

The weight loss response to anti-obesity drugs is highly variable and poorly understood, which does not allow us to know, in advance, in which subjects the drug will be effective and in which it will not. The objective of this study was to explore the body weight reduction in kilograms in the first month (1mo-BWRkg) and the development of tolerance as predictors of 6-month efficacy for treatment with 1 mg mazindol twice a day. One hundred ninety-six obese subjects were individually or jointly analyzed. Approximately 60% of subjects developed tolerance to mazindol and achieved increasing proportional levels of 6-month efficacy according to 1mo-BWRkg intervals (<1 kg, 1 to <2 kg, 2 to <4 kg and ≥4 kg). Both moT and 1mo-BWRkg were significantly correlated with the mean percentage body weight reduction (BWR%) after 6-months of treatment. The qualitative analysis of both predictors on the progressive efficacy of mazindol was used to classify patients according to expected efficacy (inefficient, slightly effective, partially effective, or fully effective), based on the mean percentage efficacy and the number of subjects reaching a BWR% of <5%, 5 to <10%, 10 to <15% or ≥15%. In conclusion, combined 1mo-BWRkg and moT were early predictors for the progressive efficacy of 6-month mazindol anti-obesity therapy. This finding represents progress in predictive, preventive, and personalized medicine which could serve for estimating the expectations of individual efficacy with the use of the drug. and highlights the basic principle of personalized medicine, “one size does not fit all”.

## 1. Introduction

There is evidence that weight loss in obese patients can reduce premature mortality from all causes, including those of a cardiovascular and oncologic origin [1]. Furthermore, it can prevent or ameliorate conditions, such as high blood pressure, diabetes mellitus, dyslipidemia, hyperglycemia, osteoarthritis, stress urinary incontinence, and gastroesophageal reflux disease, among others [2].

There is limited adoption of behavioral modifications, diet, and exercise as sole strategies for achieving weight loss. Therefore, current guidelines recommend adding pharmacological therapy for patients with a body mass index (BMI) > 30 kg/m^2^ or a BMI > 27 kg/m^2^ plus complications, such as type 2 diabetes (T2D), hypertension and dyslipidemia [3,4,5,6,7]. However, appetite suppressants have some properties in common which lead to heterogeneous results in patients. They produce a limited reduction in body weight, with a body weight plateau usually reached within 6-months of starting active treatment. Furthermore, as soon as the drug is withdrawn, subjects regain the body weight lost (i.e., an inability to maintain a reduced body weight in the absence of the drug) [8,9]. Thus, guidelines for obesity management highlight that if no clinical improvement is observed (at least 5% body weight reduction relative to baseline weight after 12 weeks of treatment with one anti-obesity medication), alternative anti-obesity drugs or increases in dosing can be considered.

The expectations of 3-month efficacy are usually high due to the emotional and economic exhaustion of obesity. Therefore, early indicators of the potential efficacy of available anti-obesity drugs have largely been sought [10]. Recently, our group described acceptable correlations between body weight loss in kilograms after one month (1mo-BWRkg) of treatment with phentermine, and the month of development of tolerance (moT) to the weight-reducing effect of the drug, with the 6-month anti-obesity efficacy of phentermine [11].

Mazindol is an old drug, still marketed as a medication for short term (three months) weight loss in countries, such as Mexico, Central America and Japan, among others. It induces weight loss through direct suppression of the appetite centers located in the lateral hypothalamus by stimulating the release of catecholamines [12].

The current study was carried out to evaluate 1mo-BWRkg and moT as combined early predictors of the 6-month percentage body weight reduction (BWR%) efficacy of mazindol.

## 2. Materials and Methods

### 2.1. Study Design

In this prospective, phase IV open-label study, 196 obese patients aged ≥ 18 years with a BMI ≥ 30 kg/m^2^ were recruited to evaluate the six-month efficacy and safety of oral administration of 1 mg mazindol twice a day. Exclusion criteria were hypersensitivity to sympathomimetic drugs, patients receiving other anti-obesity drugs, and patients diagnosed with any lung, kidney, liver, endocrine, or cardiac disease, psychiatric disorders, history of substance abuse, or pregnancy. The baseline health status of patients was determined by medical history taking and clinical examination. Patients received medical advice and were instructed to follow a diet of 1500 kilocalories, as well as physical activity for 20 min per day. They received various menus ranging from 1400 to 1600 kcal and were instructed to record daily compliance with a diet and exercise schedule.

The first tablet of 1 mg mazindol was prescribed orally 20 min before breakfast and the second tablet, 20 min before lunch. Body weight and body mass index (BMI) were evaluated at monthly visits between months 0 and 6. Baseline fasting plasmatic glucose (FPG), triglycerides (TG), total cholesterol, and thyroid stimulant hormone (TSH) levels were measured at enrollment in the study. Safety was evaluated at every visit by directed anamnesis and physical examination, and by reviewing the patient’s diary. In addition, patients were required to return the medication bottle at each visit to determine adherence to treatment.

At every visit, patients were required to empty their bladder upon arrival, subsequently dressing in a hospital gown, after which body weight was determined with a calibrated scale. Height was determined with the patient standing with the heels together, and the buttocks, shoulders, and head in contact with the stadiometer. Measurements of systolic blood pressure (SBP) and diastolic blood pressure (DBP) were obtained with an electronic sphygmomanometer. FPG, TG, total cholesterol and TSH levels were determined by standard laboratory blood chemistry measurements. The study was carried out between 8 June 2015 and 26 September 2016, at Instituto Nacional de Enfermedades Respiratorias (INER) “Ismael Cosio Villegas”.

The body weight loss in kilograms (BWRkg) was calculated by subtracting the weight at any given time (month) in the six-month study period from the weight at the beginning of the study. Percentage body weight reduction (BWR%) was calculated with the percentage of weight lost for every month with respect to baseline weight. Patients were first grouped according to their 1mo-BWRkg as follows: <1 kg, 1 to <2 kg, 2 to <3 kg, 3 to <4 kg, 4 to <5 kg, 5 to <6 kg, 6 to <7 kg, and ≥7 kg. These groups were further categorized into the following four ranges of 1mo-BWRkg: <1 kg, 1 to <2 kg, 2 to <4 kg and ≥4 kg, according to body weight reduction trends in the sixth month. The final efficacy of treatment was evaluated with the percentage of patients who achieved a BWR% of ≥5%, ≥10%, or ≥15%, and the mean (SD) BWR%.

Tolerance to mazindol’s weight-reducing effect was defined as the absence of body weight loss for any given month with respect to the prior month. The patients were classified into six groups according to the month when they developed tolerance to mazindol: month 2 (2moT), month 3 (3moT), month 4 (4moT), month 5 (5moT), month 6 (6moT), or no tolerance (NT). Early tolerance was considered when tolerance to the weight-reducing effect occurred between the 2nd and 3rd months, whereas late tolerance was considered if it occurred between months 4 and 6.

Based on the impact of these two variables on the efficacy of mazindol, 1mo-BWRkg groups (<1 kg, 1 to 2 kg, 2 to <4 kg and ≥4 kg) were subdivided into subgroups, according to moT for further analyses.

### 2.2. Data Analysis

Descriptive data for quantitative variables are presented as mean with standard deviation (SD) and as frequencies with percentages for qualitative variables. Data from subjects with at least 80% treatment adherence were grouped by 1mo-BWRkg or moT and mean monthly BWR%. Several subgroups of subjects were formed for further analyses.

The correlation between 1mo-BWRkg or moT, and 6-month BWR% was calculated with Pearson’s correlation test or Spearman’s correlation test. Data are presented as Pearson (r) or Rho (ρ) correlation coefficients with their statistical significance values (p). Qualitative comparisons were made using the chi-square test or Fisher’s exact test.

Based on the impact of the two previously analyzed variables on the efficacy of mazindol, 1mo-BWRkg groups (<1 kg, 1 to 2 kg, 2 to <4 kg and ≥4 kg) were divided into subgroups for qualitative analysis, according to the month of development of tolerance: 2moT, 3moT, 4moT, 5moT, 6moT or NT.

Differences were considered statistically significant with a bilateral *p* < 0.05. Statistical analyses were carried out in the SPSS v. 22.0 software (New York, NY, USA). Graphics were created in GraphPad Prism v.9.0.2 (San Diego, CA, USA).

## 3. Results

### 3.1. Demographic Data

Most patients were women (82.6%), aged 38.0 (SD: 9.1) years and with class 1 (66.8%) or class 2 (32.1%) obesity. Mean blood pressure, fasting glycemia, total cholesterol, and thyroid stimulant hormone levels were within normal reference values, although mean triglyceride levels were slightly above reference values, Table 1. The average self-reported adherence to diet and exercise was ~60%. Nonetheless, many patients declared having forgotten to complete the adherence questionnaires. A 94.1% average adherence to the drug was recorded.

### 3.2. Efficacy of Mazindol Treatment

**Figure 1:**
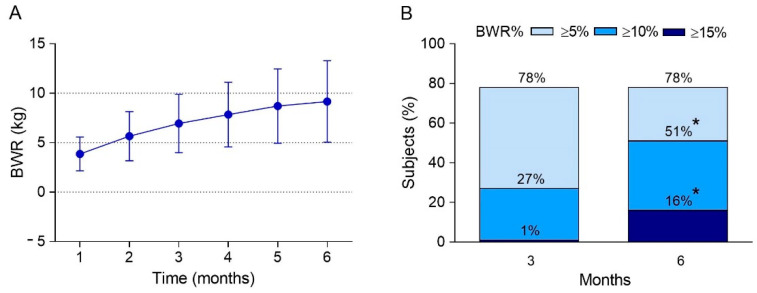
Six-month trends of mazindol effect on body weight reduction (BWRkg) of 196 subjects. Each point represents the mean and standard deviation (Panel **A**). Three- and six-month percentage of subjects achieving a ≥5%, ≥10% and ≥15% body weight reduction (Panel **B**). Abbreviations. BWR%: body weight reduction in percentage; kg: kilograms, SD: standard deviation, * Significantly different by Fisher’s test (*p* < 0.05).

Mazindol induced a mean BWR of 6.9 (SD: 2.9) and 9.1 (SD: 4.1) kg, corresponding to a mean BWR% of 8.0 (SD: 3.2) and 10.7 (SD: 4.6) percent, after 3- and 6-months, respectively. (Figure 1A). Regarding BWR% after 3-months, 78%, 27% and 1% of subjects reached ≥5%, ≥10%, and ≥15%, respectively. The percentage of patients achieving a BWR% ≥ 5% by month 6 was similar, whereas the proportions significantly increased for the ≥10% (51%) and ≥15% (16%) thresholds (*p* < 0.05) (Figure 1B).

### 3.3. Impact of 1mo-BWRkg and Development of Tolerance on the Efficacy of Mazindol

**Figure 2:**
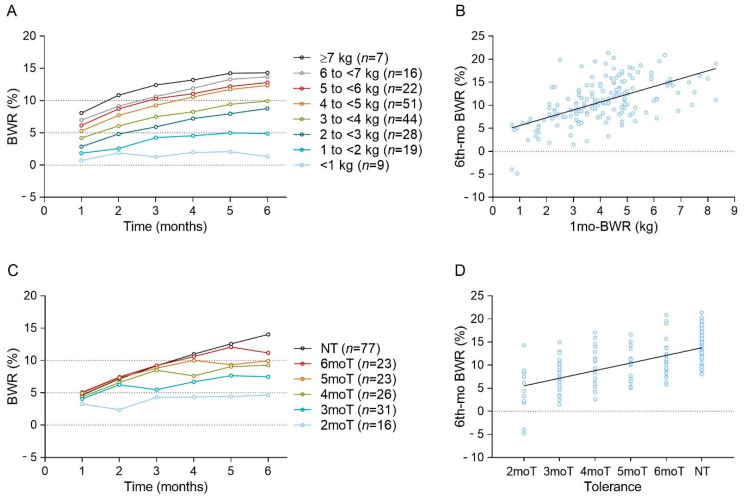
Six-month trends of mazindol effect on the body weight reduction according to body weight loss in kilograms during the first month (1mo-BWRkg, Panel **A**) and month of tolerance (Panel **C**). In Panels A and C each point represents the mean. Standard deviations were omitted to allow visualization of overlapping traces. Correlation curves between 1mo-BWRkg (Panel **B**) or month of development of tolerance to mazindol (Panel **D**), with 6mo-BWR%.

The classification of patients according to 1mo-BWRkg exhibited an apparent relationship with the subsequent efficacy of mazindol in terms of mean BWR% (Figure 2A). Subjects with a 1mo-BWRkg of <1 kg, 1 to <2 kg, 2 to <4 kg and ≥4 kg seemed to reach a mean BWR% < 5%, 5%, ~10%, and >15%, respectively. The correlation curve between 1mo-BWRkg and 6mo-BWR% showed a significant Pearson’s r coefficient of 0.58 (*p* < 0.05) (Figure 2B). In addition, the classification of patients according to the month of development of tolerance showed that approximately 60% of subjects suffered this phenomenon anytime during the 6-month treatment period (Figure 2C). The correlation curves between moT and 6th-mo BWR% also showed a significant Rho coefficient of 0.61 by Spearman´s correlation test (*p* < 0.05) (Figure 2D).

### 3.4. Impact of Early Tolerance and 1mo-BWRkg on BWR% Efficacy at Month 6

Table 2 describes the percentage of subjects who achieved a BWR% of ≥5%, ≥10%, or ≥15% at months 3 and 6, as well as the mean (SD) percentage body weight reduction at 6-months (6mo-BWR%), according to the month of development of tolerance (2moT or 3moT), no tolerance after three months (3moNT), and 1mo-BWRkg. The minimum value of subjects who advanced to the next level is shown alongside the 1mo-BWRkg interval. In addition, Table 2 shows the number of withdrawals before completing 6-months of treatment (WD) and the number of subjects in whom treatment was ineffective (considered as BWR% < 5%).

The efficacy profile tended to be proportional to 1mo-BWRkg and moT. Four levels of minimum target BWR% were arbitrarily built by setting ≥5% as the reference. **Inefficient** after 6-months of treatment was considered when the target efficacy (≥5%) was not achieved by any of the subjects (2moT plus 1mo-BWRkg < 2 kg, and 3moT plus 1mo-BWRkg < 1 kg, highlighted in red). This occurred in a small number of patients (*n* = 7). **Slightly effective** (*n* = 18) was considered when the target efficacy (≥5%) was reached (although not necessarily in all subjects by month 6) and the average 6mo-BWR% was at the efficacy range BWR% 5 to <7% (2moT plus 1mo-BWRkg of 2 to <4 kg, 3moT plus 1mo-BWRkg of 1 to <2 kg, or NT plus 1mo-BWRkg < 2 kg; highlighted in orange). **Partially effective** (*n* = 9) occurred when the target efficacy (≥5%) was reached (although not necessarily in all subjects by month 6), and the average 6mo-BWR% was at the efficacy range of 7 to <10% (3moT plus 1mo-BWRkg of 2 to <4 kg or 3moT plus 1mo-BWRkg of ≥4 kg; highlighted in yellow). A limited percentage of subjects advanced through the next efficacy level (≥10%). Lastly, **fully effective** (*n* = 143) was set as 100% of the subjects reaching the efficacy target (≥5%) and an additional percentage of subjects achieving the next efficacy levels of ≥10 or ≥15% (2moT plus a 1mo-BWRkg ≥ 4 kg or NT with 1mo-BWRkg > 2 kg; highlighted in green). Reaching the higher efficacy level tended to occur in subjects within the highest 1mo-BWRkg range. No evident relevant efficacy or ineffectiveness trends were observed in association with baseline blood glucose, TSH, TGC, cholesterol, or LDL cholesterol.

### 3.5. Impact of Late Tolerance and 1mo-BWR on BWR% Efficacy at Month 6

Table 3 describes the same parameters shown in the previous table, in 136 subjects who did not develop tolerance during the first 3-months of treatment and who completed the 6-month follow-up period. The development of late tolerance over months 4 to 6 apparently had a lower impact on the 6-month efficacy of mazindol than early tolerance. In this group, mainly two levels of efficacy of those described above were observed.

Nine subjects showed slight effectiveness (4moT plus 1mo-BWRkg of 1 to <2 kg, 5moT plus 1mo-BWRkg < 2 kg, or 6moT plus 1mo-BWRkg < 1 kg, highlighted in orange) and 125 subjects achieved full effectiveness (highlighted in green). Only a limited percentage of subjects with 6moT or NT plus a 1mo-BWRkg of 2 to <4 kg subjects reached the highest ≥15% efficacy level after 6-months of treatment. In addition, there was one withdrawal in the first month within the NT plus a 1mo-BWRkg of 1 to <2 kg group. Conversely, in subjects with a 1mo-BWRkg ≥ 4 kg, there was a tendency towards a proportional BWR% efficacy of ≥10% and ≥15% according to the month of development of tolerance.

### 3.6. Safety of Mazindol

A total of 889 adverse events (AEs) were reported for 196 patients receiving 1 mg mazindol twice a day, during the 6-month follow-up period. Table 4 shows the 15 most frequently reported AEs, including mouth dryness, headache, constipation, fatigue, and insomnia. Most AEs were mild (773) and only 116—mainly headache and constipation—were moderate. Interestingly, 16 out of 196 subjects did not report AEs; eight of them were mostly in the NT category and had varying levels of 1mo-BWRkg. The other eight subjects completed 6-months of treatment with varying degrees of 1mo-BWRkg with two of them having 2moT or 3moT; two, 5moT or 6moT and four, NT.

## 4. Discussion

The current study aimed to evaluate the trends in body weight reduction by the administration of 1 mg mazindol twice daily for 6-months, assessing predictors, such as weight loss during the first month and the month of development of tolerance to the weight-reducing effect of mazindol.

We found that daily oral administration of mazindol in a sample of subjects with class 1 and 2 obesity induced a mean weight loss of 6.9 kg and 9.1 kg after 3- and 6-months of treatment, respectively, corresponding to BWR% of 8.0% and 10.7%, respectively. Although there are no other clinical studies with a similar design or populations that could be used to contrast our findings, clinical trials of short-term treatment—median 12 weeks—have reported a mean body weight loss of 5.2 kg [13], whereas trials of long-term treatment—up to 60 weeks—have shown an average reduction of 6.8 kg [14]. The wide interindividual variability observed in our study is in line with general drug therapy in patients with obesity [15].

Mazindol is a centrally acting anorexigenic agent that is not derived from β-phenylethylamines, so it does not share the side effects and potential for addiction and abuse of amphetamine-group compounds [16]. The main mechanism of action proposed to explain the anorexigenic effect of mazindol seems to be related to the stimulation of β-adrenergic receptors [17] and the direct suppression of glucose-sensitive neurons that it exerts on the appetite centers located in the lateral hypothalamus [12,18], translating into less secretion of gastric acid and decreased appetite [19]. Additionally, mazindol reduces glucose absorption in the small intestine [20], blocked insulin secretion from the ventromedial hypothalamus [21] and augments locomotor activity, which could enhance energy expenditure [22].

In our study, 78% of subjects treated with mazindol achieved BWR% efficacy levels greater than or equal to 5%, including 28% of subjects that achieved values ≥10% after 3-months. These reductions tended to be maintained by month 6, with significant increases in weight loss that would support the administration of mazindol for 6-months, according to current guidelines for the management of obesity. In line with our results, a study reported that only 79.5% of subjects treated with mazindol (0.5–3 mg/day) experienced body weight loss [12]. These values are similar to the percentage of subjects that report appetite suppression with mazindol treatment (70–90%) [12,14]. Thus, mazindol is likely ineffective in nearly 20% of subjects.

Additionally, there was a clear trend between 1mo-BWRkg and moT with a progressive weight reduction induced by mazindol by month 6, which was confirmed by the existence of significant correlations in both cases. Furthermore, approximately 60% of subjects developed tolerance to the weight-lowering effect of mazindol; 24% of them by months 2 and 3, and 37%, after 4–6 months of treatment. In this regard, it has been reported that mazindol treatment is able to maintain body weight loss for 1 year in 53.3% of subjects after a very low caloric diet [14]. In our study, we found a progressive mean BWR% efficacy in relation to 1mo-BWRkg intervals: <1 kg, 1 to <2 kg, 2 to < 4 kg, and ≥4 kg, similar to what has been observed by our group in patients treated with 30 mg phentermine [11]. These results highlight the usefulness of the long-term use of mazindol in a subgroup of obese subjects.

The combined qualitative analysis of the development of tolerance during the first 3-months and the 1mo-BWRkg showed that mazindol is not effective in patients with 2moT plus 1mo-BWRkg of <2 kg or in those with 3moT plus 1mo-BWRkg <1 kg after 3- and 6-months of treatment, whereas the best efficacy levels were observed in subjects with 2moT or 3moT plus a 1mo-BWRkg ≥ 4 kg. The rest of the subgroups reached intermediate efficacy levels. Regarding the NT group, proportionally increasing levels of efficacy were observed for all 1mo-BWR% intervals. Furthermore, the best efficacy levels were found in the group of subjects whose 1mo-BWRkg was in the upper level of the categories 2 to <4 kg and ≥4 kg. In addition, blood glucose, TSH, TGC, cholesterol, or LDL cholesterol were not associated with efficacy levels.

The analysis of late tolerance to mazindol (four to six months) showed that this occurred in subjects with a 1mo-BWRkg < 2 kg, having a lower impact on the 6-month efficacy of mazindol, still reaching intermediate efficacy levels. In contrast, the rest of the subgroups reached acceptable and proportionally increasing levels of efficacy according to all 1mo-BWRkg and moT intervals considered.

To the best of our knowledge, this is the first analysis of the impact of the development of tolerance and 1mo-BWRkg on the 3-month and 6-month efficacy of mazindol in the treatment of obesity. These findings were similar to those observed with 30 mg phentermine [11]. Beyond the recommendations described in the current obesity treatment guidelines, we consider that our data shown in Table 2 and Table 3, including 1mo-BWRkg, moT, mean BWR%, and percentage of subjects achieving specific levels of efficacy by months three and six, represent recent progress in predictive, preventive, and personalized medicine; they allow an earlier qualitative evaluation and predictive diagnostic of the progressive potential efficacy of the drug, as well as to modify the treatment strategy earlier according to specific and individual weight reduction targets. This finding adds to the interest of various authors in defining variables that allow establishing the bases for understanding the great interindividual variability observed in the treatment of a disease of multifactorial origin, such as obesity and highlights the necessity to impulse individualized treatment [23,24,25].

Although an important number of adverse events were reported during the 6-month treatment with mazindol, these were mostly mild in intensity, including dry mouth, headache, constipation, and fatigue. These adverse events were expected and they have been previously reported in other studies [12,14,26]. Interestingly, the frequency of adverse events was not related to the development of tolerance to mazindol, which could suggest that tolerance could not be exclusively due to pharmacological aspects but to intrinsic homeostatic resistance to body weight reduction [9].

The limitations of this study include that analyses were performed in a small sample of predominantly obese subjects with class 1 and 2 obesity of Mexican women younger than 45 years, which could limit the extrapolation of our results to other populations. Despite the predictive potential of 1mo-BWRkg and moT on the efficacy of mazindol being only qualitative, with a suboptimal representation of subgroups, we consider that these results could be useful to reduce the uncertainty about the progressive efficacy of the treatment of obesity for 6-months with mazindol.

Furthermore, studying the few anti-obesity drugs currently available through similar approaches would allow us to have better characterizations of agents with different mechanisms of action. In order to diminish or reverse tolerance to anti-obesity drugs, the addition of drugs, such as metformin, orlistat, or others could possibly serve this purpose, which could further inform clinical practice guidelines for the treatment of obesity.

## 5. Conclusions

In conclusion, the combination of 1mo-BWRkg and moT could be useful predictors of the expectations of mazindol efficacy during a 6-month treatment period, always in accordance with the approved indications for use in each country where mazindol is available. In the context of predictive, preventive and personalized medicine principles, these predictors would allow an individual qualitative evaluation of the progressive potential efficacy of the drug, as well as to modify the treatment strategy earlier according to weight reduction targets.

## Figures and Tables

**Table 1:** Baseline characteristics of subjects in this study.

Parameter	Total Sample (*n* = 196)	Reference Value
Women, *n* (%)	162 (82.6)	-
Age, years (SD)	38.0 ± 9.1	-
Weight, kg (SD)	85.8 (10.6)	-
Body Mass Index, kg/m^2^ (SD)	33.9 (2.8)	-
Class 1 obesity, *n* (%)	131 (66.8)	30–34.9 kg/m^2^
Class 2 obesity, *n* (%)	63 (32.1)	35–39.9 kg/m^2^
Class 3 obesity, *n* (%)	2 (0.1)	≥40 kg/m^2^
SBP, mmHg (SD)	112.4 (10.1)	<120 mmHg
DBP, mmHg (SD)	69.6 (7.9)	<80 mmHg
Fasting glycemia, mg/dL (SD)	93.8 (9.8)	75 ≤ 99 mg/dL
Triglycerides, mg/dL (SD)	180.5 (82.6)	<150 mg/dL
Total Cholesterol, mg/dL (SD)	185.7 (36.64)	<200 mg/dL
TSH, μUI/mL (SD)	2.6 (1.7)	0.34–5.6 μUI/mL

Data are expressed as mean (SD) or as frequencies (%). BMI: Body mass index; SBP: Systolic blood pressure; DBP: Diastolic blood pressure; TSH: Thyroid stimulant hormone; SD: Standard deviation.

**Table 2:** Mazindol efficacy expectations based on early tolerance and 1mo-BWRkg.

1mo-BWR		BWR	3 Months	6 Months	6mo-BWR (%)		WD	Ineffective
kg	*n*	%	*n* (%)	*n* (%)	Mean (SD)	*n*	*n*	*n*
**2moT**								
**<1**	2	≥5	0 (0)	0 (0)	4.3 (0.6)	2	0	2
**1 to <2**	2	≥5	0 (0)	0 (0)	−3.8 (0.7)	2	0	2
**2 to <4**	4	≥5	1 (25)	2 (50)	−6.8 (0.8)	2	0	2
**≥4**	4	≥5	4 (100)	4 (100)	−8.2 (0.6)	3	0	0
*(Min: 6.5)*		≥10	1 (25)	1 (25)	−14.3	1		
**3moT**								
**<1**	3	≥5	0 (0)	0 (0)	−4.6	1	2	1
**1 to <2**	4	≥5	1 (25)	2 (67)	−5.9 (1.1)	2	1	1
**2 to <4**	9	≥5	4 (44)	6 (75)	−8.3 (1.3)	4	1	2
*(Min: 3.2)*		≥10	0 (0)	2 (25)	−11.6 (1.7)	2		
**≥ 4**	15	≥5	15 (100)	14 (93)	−7.2 (1.8)	10	0	1
*(Min: 4.9)*		≥10	1 (7)	4 (27)	−12.2 (2.0)	4		
**3moNT**								
**<1**	2	≥5	0 (0)	2 (100)	−5.3 (0.5)	2	0	0
**1 to <2**	8	≥5	4 (50)	5 (71)	−5.2 (1.4)	5	1	2
**2 to <4**	53	≥5	50 (94)	50 (100)	−8.0 (1.3)	29	3	0
*(Min: 3.1)*		≥10	6 (11)	21 (42)	−12.0 (1.4)	17		
*(Min: 3.2)*		≥15	0 (0)	4 (8)	−18.4 (1.6)	4		
**≥4**	74	≥5	74 (100)	69 (100)	−8.0 (1.3)	6	5	0
*(Min: 4.0)*		≥10	42 (57)	63 (91)	−12.4 (1.5)	35		
*(Min: 4.2)*		≥15	2 (3)	28 (41)	−17.2 (1.7)	28		

Data are expressed as the percentage of subjects who achieved a BWR% of 5 to <10 %, 10 to <15%, or ≥15% after 3- and 6- months of treatment, or as the mean (SD) percentage body weight reduction by month 6 (6mo-BWR%) according to the month of development of tolerance (2moT or 3moT) or absence of tolerance (NT) and the body weight loss in kilograms after one month (1mo-BWRkg). Abbreviations: *n* = number of patients, WD = withdrawals before completing 6-months of treatment. Min = minimum reduction observed in the 1mo-BWRkg to reach 10 to <15 kg or ≥15 Kg at month 6.

**Table 3:** Mazindol efficacy expectations based on late tolerance and 1mo-BWRkg.

1mo-BWR		BWR	3 Months	6 Months	6mo-BWR (%)		WD	Ineffective
kg	*n*	%	*n* (%)	*n* (%)	Mean (SD)	*n*	*n*	*n*
**4moT**								
**1 to <2**	4	≥5	2 (50)	2 (50)	−6.1 (0.0)	2	0	2
**2 to <4**	11	≥5	11 (100)	10 (100)	−7.0 (1.3)	8	1	0
*(Min: 2.3)*		≥10	1 (9)	2 (20)	−12.7 (1.7)	2		
**≥4**	11	≥5	11 (100)	10 (100)	−9.0 (0.5)	3	1	0
*(Min: 4.0)*		≥10	6 (55)	7 (70)	−12.1 (1.5)	5		
*(Min: 4.4)*		≥15	0 (0)	2 (20)	−16.4 (0.8)	2		
**5moT**								
**<1**	1	≥5	0 (0)	1 (100)	−5.0	1	0	0
**1 to <2**	3	≥5	2 (67)	3 (100)	−5.8 (1.0)	3	0	0
**2 to <4**	4	≥5	4 (100)	4 (100)	−8.4 (1.3)	3	0	0
*(Min: 3.9)*		≥10	1 (25)	1 (25)	−11.0	1		
**≥4**	15	≥5	15 (100)	14 (100)	−6.4 (0.0)	2	1	0
*(Min: 4.1)*		≥10	7 (47)	12 (86)	−11.5 (1.3)	10		
*(Min: 4.5)*		≥15	0 (0)	2 (14)	−15.8 (1.0)	2		
**6moT**								
**<1**	1	≥5	0 (0)	1 (100)	−5.7	1	0	0
**2 to <4**	11	≥5	10 (91)	11 (100)	−7.8 (1.4)	8	0	0
*(Min: 3.1)*		≥10	1 (9)	3 (27)	−13.6 (5.0)	2		
*(Min: 3.8)*		≥15	0 (0)	1 (9)	−19.4	1		
**≥4**	11	≥5	11 (100)	11 (100)	−8.2	1	0	0
*(Min: 4.3)*		≥10	8 (72)	10 (91)	−11.9 (1.4)	7		
*(Min: 6.4)*		≥15	2 (18)	3 (27)	−18.5 (2.6)	3		
**6moNT**								
**1 to <2**	1	≥5	0 (0)			0	1	0
**2 to <4**	27	≥5	25 (93)	25 (100)	−8.8 (0.7)	10	2	0
*(Min: 2.1)*		≥10	3 (11)	15 (60)	−12.1 (1.5)	12		
*(Min: 2.5)*		≥15	0 (0)	3 (12)	−18.1 (1.8)	3		
**≥4**	37	≥5	37 (100)	34 (100)			3	0
*(Min: 4.0)*		≥10	23 (62)	34 (100)	−13.4 (1.1)	13		
*(Min: 4.2)*		≥15	0 (0)	21 (62)	−17.3 (1.7)	21		

Data are expressed as the percentage of subjects who achieved a BWR% of 5 to <10 %, 10 to <15%, or ≥15% after 3- and 6-months of treatment, or as the mean (SD) percentage body weight reduction by month 6 (6mo-BWR%) according to the month of development of tolerance (4moT, 5moT or 6moT) or absence of tolerance (NT) and the body weight loss in kilograms after one month (1mo-BWRkg). Abbreviations: *n* = number of patients, WD = withdrawals before completing 6-months of treatment. Min = minimum reduction observed in the 1mo-BWRkg to reach 10 to <15 kg or ≥15 Kg at month 6.

**Table 4:** Main adverse events reported by patients with obesity who received mazindol for 6-months.

Adverse Event	Total (%)	Mild (%)	Moderate (%)
Dry mouth	99 (11.1)	99 (11.1)	-
Headache	98 (11.0)	70 (7.9)	28 (3.1)
Constipation	66 (7.4)	44 (4.9)	22 (2.5)
Fatigue	53 (6.0)	53 (6.0)	-
Insomnia	47 (5.3)	45 (5.1)	2 (0.2)
Hyperhidrosis	46 (5.2)	46 (5.2)	-
Thirst	41 (4.6)	41 (4.6)	-
Nausea	38 (4.3)	37 (4.2)	1 (0.1)
Anxiety	35 (3.9)	35 (3.9)	-
Chills	35 (3.9)	34 (3.8)	1 (0.1)
Drowsiness	32 (3.6)	31 (3.5)	1 (0.1)
Dysgeusia	23 (2.6)	23 (2.6)	-
Dyspepsia	19 (2.1)	13 (1.5)	6 (0.7)
Tachycardia	15 (1.7)	6 (0.7)	9 (1.0)
Abdominal pain	14 (1.6)	9 (1.0)	5 (0.6)
Others	228 (25.7)	187 (21.0)	41 (4.6)
**Total**	**889 (100%)**	**773 (87.0%)**	**116 (13.0%)**

## Data Availability

Raw data were generated at the National Institute of Respiratory Diseases, Ismael Cosio Villegas. Data supporting the findings of this study are available from the corresponding author on reasonable request.
